# Liability of Hospitals

**Published:** 1909-07-24

**Authors:** 


### Liability of Hospitals.

### ST. BARTHOLOMEW'S CASE: APPEAL.

In the Court of Appeal on July 13, before the Master of the Rolls and Lords Justices Farwell and Kennedy, the case of "Hillyer *v.* Corporation of the City of London" was reconsidered. As reported in The Hospital at the time and since commented upon, the defendants were sued, as the Governors of St. Bartholomew's Hospital, by Mr. William H. Hillyer, a member of the medical profession. The plaintiff alleged that while undergoing an examination under anæsthetics on the operating-table of the hospital his left arm, by the negligence of the defendants or that of their servants, was allowed to come into contact with the hot-water tins used for heating the table, and as a result he was paralysed in that arm, while the right arm was also injured. The defendants denied liability, and pleaded that, if it was not an inevitable accident, there was no relation of master and servant existing between themselves and those engaged in the examination which made them responsible in law.—Mr. J. B. Matthews, in support of the appeal by the plaintiff against the verdict of Mr. Justice Grantham, who, it will be remembered, refused to send the case to a jury, said that at any rate there was a *primâ-facie* case made out by the plaintiff fit for the consideration of the jury. The judge, therefore, ought not to have ruled as he had done, and the plaintiff was entitled to a new trial. Counsel referred at considerable length to the evidence, and after Mr. McCall, K.C., and Mr. H. C. Marks had pleaded in support of the judgment, Mr. Matthews replied. —The Master of the Rolls (after consulting with the Lords Justices) finally said: "We think we must take time to consider this case, as the question of the respondents' liability is a very important one."

We received some little time ago from Messrs. Coleman and Co., of Norwich, contractors to the Royal Army Medical Corps, a complete set of their useful "Wincarnis" Motor and Cyclists' Sectional Maps of England and Wales. Now that the holiday season is upon us we may appropriately draw attention to the series, which consists of 16 separate maps, each 9 inches by 6½ inches in size, and clearly printed on stout paper. These convenient little sectional maps can be obtained by readers on application to the "Wincarnis" works. Any one of the 16 sections will be sent on receipt of a postcard, and the complete set will be sent to any address on receipt of two penny stamps. The scale is 10 miles to the inch, and all the principal roads are clearly marked in red, while railway lines are also shown. The series reflects credit upon Messrs. Fletcher and Son, Ltd., map-lithographers, also of Norwich, whose work it is.

The rate of annual subscriptions to The Hospital, either direct from the Office or through agents, is :—

| | |
|---|---|
| For the United Kingdom ...... ... ... | 15s. a year. |
| For the Colonies and Abroad ... ... ... | 17s. ,, |
| For the United Kingdom (with Insurance Policy) ... ... ... ... ... ... ... ... | £1 ,, |

inclusive of postage in each case.

THE HOSPITAL July 24, 1909.

*Name* ..............................

*Address* ..............................

..............................

This Coupon must accompany manuscript or contributions intended for The Hospital.

